# Observations on Accouchements, from Cases Occurring in the Lying-In Hospital at Strasburg

**Published:** 1817-03

**Authors:** 

**Affiliations:** first Anatomist of the Faculty of Medicine, and first Physician-Accoucheur of the Civil Hospital of Strasburg.


*For the London Medical and Physical Journal.*

*Observations on Accouchements, from Cases occurring in the Lying-in Hospital at Strasburg;* by Dr. LOBSTEIN, first Anatomist of the Faculty of Medicine, and first Physician-Accoucheur of the Civil Hospital of Strasburg.—Communicated to the Society of Medical Emulation of Paris.

*Cæsarean Operation.*

I HAVE to render an account of three Cæsarean operations performed almost immediately after the death of the mothers. In truth, these operations do not differ from the opening of a body, excepting that it is necessary to proceed in the same manner, and to take the same precautions as if it were in a living person.

1. The first died at the hospital, in the eighth month of her pregnancy, of a dropsy of the chest, arising from a neglected glected quartan fever. The section was made in the *linea alba.* The fœtus, which was well situated for a natural accouchement, no longer existed, though a quarter of an hour had not elapsed from the death of the mother. The matrix contracted in such a manner, that, after having extracted the infant by the wound made on the anterior coat of this viscus, it was impossible to introduce the hand to seek the placenta: I therefore left it, and injected the matrix, to preserve it in the Cabinet of the Faculty. I perfectly succeeded in filling the vessels of the matrix, as well as the parenchyma and the cell of the placenta; but nothing would penetrate in the umbilical vessels of the fœtus.

Not only in this case, but also in a more recent one, I observed that the death of the child almost immediately followed that of the mother.

2. I was called, on the 4th of June, 1815, to attend Mary Salome Burger, who, I was told, was pregnant for the first time, and had been in convulsions twenty-four hours. She had expired five minutes before my arrival. She was in the ninth month of her pregnancy. The section was made in the linea alba. The uterus being opened, and the membranes pierced, I withdrew the fœtus, which had the thighs upward and the head downwards, or in the first position of *Baudelocque.* Although the body of the woman, as well as that of the child, was quite warm, the latter gave no signs of life: there was no pulsation in the umbilical cord, and the ordinary succours were administered in vain. The incision of the uterus furnished a large quantity of blood, which flowed *en masse.* The matrix, too, contracted, as in the preceding case; but I observed no peristaltic motion in the intestines, which escaped through the wounds of the integuments.

It may be said, perhaps, that the convulsions of the mother determined the death of the child. It may be; but I have witnessed many cases of lively healthy children, where the mothers have experienced, during pregnancy and labour, strong attacks of epilepsy.

3. The third case was in a woman who died at the hospital with all the signs of a rupture of the matrix. The section being made as before, and the infant extracted, I found—1, a quantity of extravasated blood in the hypogastric region; 2, a rupture of the superior part of the posterior coat of the vagina, as well as in the inferior part on the same side: this orifice was large enough to admit the introduction of the hand; a portion of the rectum was engaged in it.

### *Extra-Uterine Pregnancy.*

It is not only in uterine pregnancy that I have been called upon to open the hypogastric region, but twice also in cases where the fœtus developed itself in the fallopian tube.

I. Eve Conrad, æt. 35, married five years, pregnant for the first time. In her third month she felt, in the left iliac region, a tumour, which was very painful. Being fatigued one day with her usual labour, a fortnight after having drank more wine than ordinary, she was attacked with vomiting, accompanied by a violent colic, the lower belly tumefied suddenly, and she died in a fainting fit.

Her sudden death creating a suspicion of her being poisoned, I was ordered to open the body. After making an incision in the abdomen, which was swelled, but had not changed colour, I found the intestines floating in blood, half fluid and half coagulated: having removed this, I examined the alimentary tube, opening it from the insertion of the œsophagus in the stomach to the left colon, without meeting with any traces of poison. Having discovered, by the introduction of the hand into this part of the body, something contrary to nature, I extracted the parts of generation, internal and external, and then found the left Falopian tube forming a tumour of two inches long and eight-tenths of an inch wide, torn on the side next the pubis. A flaky tissue, which I soon discovered to be the placenta, as it is organized in the first periods of pregnancy, was interposed between the lips of the wound. Removing these flakes a little, and floating them in clear water, I at first discovered that the coat of the tube was one-twentieth of an inch thick. I afterwards perceived a diaphanous membrane, which concealed an embryo, in which I could distinctly discover all the parts of the body. Not daring to open this membrane, for fear of losing the embryo, I preserved it in spirits of wine. I next opened the matrix, which was larger than in a state of vacuity; and, by this section, I verified the assertion of John Hunter, that the *caducous* membrane exists even in cases of extra-uterine pregnancy. This membrane, which I found soft and pulpy, uniformly lined the whole of the internal surface of the uterus.

The tissue of the matrix was rather softer, and more vascular, and the round ligaments rather thicker, than in a state of vacuity. The spermatic vessels appeared larger, and the veins were gorged with blood. The left ovarium contained a yellow body *(corpus luteum)*, and the right, some testicles filled with a diaphanous lymph. The neck of the matrix and vagina were constituted as in women who have never had a child.

II. A woman, æt. 39, having been delivered the first time by means of forceps, found, two years afterwards, symptoms announcing announcing a second gestation, amongst which the cessation of the menses, the swelling of the breasts, nausea, vomiting, and the tooth ach, were the most striking. Towards the end of the third month, she had a considerable discharge, by which she at first rendered clotted blood, and afterwards fluid blood, and, lastly, a large quantity of serous matter. The midwife having examined her, found the matrix of the size usual in the fourth month of pregnancy; the neck of the uterus in its ordinary direction, but thicker and softer. About a month afterwards, she complained of violent pains about the right pubis, which gradually increased, and was attributed to indigestion. In the mean time, the pains assumed the character of labour-pains;—the midwife was sent for, who, after having felt, pronounced the matrix to be full; and she fancied she felt the members of a fœtus on the right side, and across the coats of the vagina. Being lifted out of bed to go to the night-table, she fainted, and frothed much at the mouth. At the same time the belly began to swell, and the surface to be discoloured, which announced an internal hæmorrhage. The patient recovered her senses, and only complained of a burning in the right iliac *fosse*, which spread to the breast by degrees. The difficulty of breathing soon became extreme, and she could no longer remain in an horizontal position. The eyes were fixed and haggard; the saliva flowed slowly from the mouth; and she expired two hours and a half after the arrival of the midwife, who never left her an instant, and from whom I received the above details.

I opened the body the same day. The lower belly, and particularly the pelvis, were found filled with blood, half fluid and half clotted. Having extracted the internal and external parts of generation, and washed them from the clotted blood which covered them, I found,—

1. The matrix more voluminous than in a state of vacuity. The coat was half an inch thick. The cavity of the neck was filled with concrete mucus, like the white of an egg, half coagulated *(le museau de touche)*,—was perfectly smooth, and rounded, with the exception of a slope on the left side, the sign of a previous accouchement. The length of the neck of the uterus was 1½ inches; the round ligaments were thicker than ordinary, and the matrix exhibited, in its cavity, the caducous membrane, but in a state of tenuity or softness; the vessels in the substance of the uterus were larger than in the state of non-pregnancy.

2. The right ovarium presented a great number of vesicles, some on the surface, and others towards the middle of the organ. The largest were of the diameter of from one to to two-tenths of an inch. Under the external membrane of the ovarium was found a corpus luteum, as well as the traces of a second. The ovarium was, in other respects, attached to the Fallopian tube of the same side.

The left ovarium had, in its surface, a vesicle fitted with a diaphanous fluid, which coagulated in spirits of wine. In the interior we found three other vesicles, of the diameter of two-tenths of an inch, also filled with lymph. In the centre of this ovarium we perceived a cavity, with a smooth coat, half an inch in diameter every way. The membrane which formed this coat was one-twentieth of an inch thick. This grand vesicle was filled with a small portion of the fibrine of the blood. There was also observed, in the substance of this ovarium, a corpus luteum, and the traces of three others.

3. The right fallopian tube was transformed into an egg-shaped tumour, the transverse diameter of which was $2\frac{3}{10}$ inches, and the congregate $1\frac{7}{10}$ inch. On the front part of this tube, dilated, was remarked a rupture of $1\frac{6}{10}$ in., where the flakes presented themselves which constitute the placenta. The coats of the tube were not every where of the same thickness. The interior of this bag did not appear to be lined with the *caducous* membrane. The embryo was distinguished across the diaphanous membranes: it was nearly $1\frac{1}{2}$ in. long; and the length of the members was such as is common to fœtuses of that age. I endeavoured to separate the *chorion* from the *amnios*, and I thereby found that the former constituted a strong dense membrane.

The left falopian tube presented nothing in particular.

